# Genome-Wide DNA Methylation Markers Associated With Metabolic Liver Cancer

**DOI:** 10.1016/j.gastha.2025.100621

**Published:** 2025-01-23

**Authors:** Samuel O. Antwi, Ampem Darko Jnr. Siaw, Sebastian M. Armasu, Jacob A. Frank, Irene K. Yan, Fowsiyo Y. Ahmed, Laura Izquierdo-Sanchez, Loreto Boix, Angela Rojas, Jesus M. Banales, Maria Reig, Per Stål, Manuel Romero Gómez, Kirk J. Wangensteen, Amit G. Singal, Lewis R. Roberts, Tushar Patel

**Affiliations:** 1 Division of Epidemiology, Department of Quantitative Health Sciences, Mayo Clinic, Jacksonville, Florida; 2 Division of Gastroenterology and Hepatology, Department of Internal Medicine, Mayo Clinic, Jacksonville, Florida; 3 Division of Clinical Trials and Biostatistics, Department of Quantitative Health Sciences, Mayo Clinic, Rochester, Minnesota; 4 Department of Cancer Biology, Mayo Clinic, Jacksonville, Florida; 5 Division of Gastroenterology and Hepatology, Department of Internal Medicine, Mayo Clinic, Rochester, Minnesota; 6 Department of Liver and Gastrointestinal Diseases, Biogipuzkoa Health Research Institute-Donostia University Hospital, University of the Basque Country (UPV/EHU), San Sebastian, Spain; 7 BCLC Group, Liver Unit, ICMDM, IDIBAPS, Hospital Clinic of Barcelona, University of Barcelona. Centro de Investigación Biomédica en Red en Enfermedades Hepáticas y Digestivas (CIBEREHD); Barcelona University, Barcelona, Spain; 8 SeLiver Group, UCM Digestive Diseases, Institute of Biomedicine of Seville (IBiS), Virgen del Rocio University Hospital/CSIC/University of Seville, Seville, Spain; 9 Hepatic and Digestive Diseases Networking Biomedical Research Centre (CIBERehd), Madrid, Spain; 10 Department of Biochemistry and Genetics, School of Sciences, University of Navarra, Pamplona, Spain; 11 Ikerbasque, Basque Foundation for Science, Bilbao, Spain; 12 Department of Upper GI Diseases, Karolinska University Hospital, Department of Medicine Huddinge, Karolinska Institutet, Stockholm, Sweden; 13 Department of Internal Medicine, University of Texas Southwestern Medical Center, Dallas, Texas; 14 Department of Transplantation, Mayo Clinic, Jacksonville, Florida

**Keywords:** Liver Cancer, HCC, MASLD, NAFLD, Metabolic Dysfunction-Associated Steatotic Liver Disease

## Abstract

**Background and Aims:**

Metabolic liver disease is the fastest-rising cause of hepatocellular carcinoma (HCC), but the underlying molecular processes that drive HCC development in the setting of metabolic perturbations are unclear. We investigated the role of aberrant DNA methylation in metabolic HCC development in a multicenter international study.

**Methods:**

We used a case-control design, frequency-matched on age, sex, and study site. Genome-wide profiling of peripheral blood leukocyte DNA was performed using the 850k EPIC array. The study sample was split 80% and 20% for training and validation. Cell type proportions were estimated from the methylation data. Differential methylation analysis was performed adjusting for cell type, generating area under the receiver-operating characteristic curves (AUC-ROC).

**Results:**

We enrolled 272 metabolic HCC patients and 316 control patients with metabolic liver disease from 6 sites. Fifty-five differentially methylated CpGs were identified; 33 hypermethylated and 22 hypomethylated in cases vs controls. The panel of 55 CpGs discriminated between the cases and controls with AUC = 0.79 (95% confidence interval [CI] = 0.71–0.87), sensitivity = 0.77 (95% CI = 0.66–0.89), and specificity = 0.74 (95% CI = 0.64–0.85). The 55-CpG classifier panel performed better than a base model that comprised age, sex, race, and diabetes mellitus (AUC = 0.65, 95% CI = 0.55–0.75; sensitivity = 0.62, 95% CI = 0.49–0.75; and specificity = 0.64, 95% CI = 0.52–0.75). A multifactorial model that combined the 55 CpGs with age, sex, race, and diabetes yielded AUC = 0.78 (95% CI = 0.70–0.86), sensitivity = 0.81 (95% CI = 0.71–0.92), and specificity = 0.67 (95% CI = 0.55–0.78).

**Conclusion:**

A panel of 55 blood leukocyte DNA methylation markers differentiates patients with metabolic HCC from control patients with benign metabolic liver disease, with a slightly higher sensitivity when combined with demographic and clinical information.

## Graphical abstract

**Figure:**
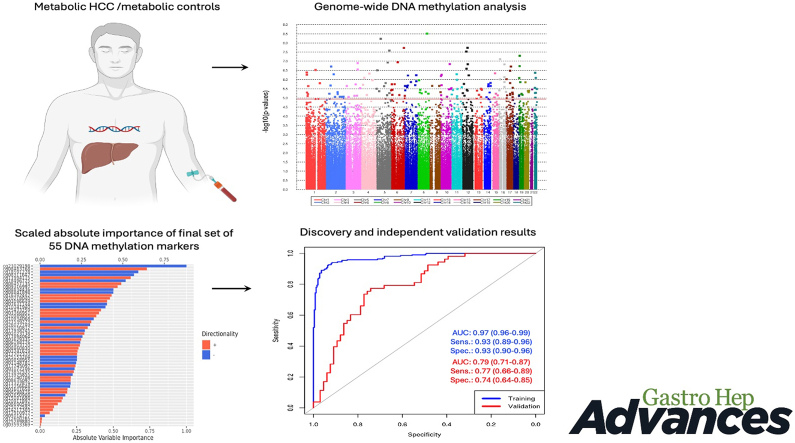

## Introduction

Metabolic liver disease is the fastest-growing cause of liver cancer and its most common type, hepatocellular carcinoma (HCC).^1^^,^^2^ Metabolic liver disease comprises metabolic dysfunction-associated steatotic liver disease (MASLD) and nonviral and nonalcoholic steatotic liver disease, and these are rapidly increasing worldwide.^2^, ^3^, ^4^ Although chronic hepatitis B and C virus (HBV and HCV) infections were for several decades the major causes of HCC, improved treatments for both HBV and HCV and increased vaccinations for HBV have shifted the burden of HCC to nonviral causes, with metabolic liver disease being the most rapidly rising cause.^5^ Metabolic HCC exhibits unique molecular processes and immune characteristics and is considered a distinct HCC type, requiring characterization of its underlying molecular signatures, including epigenome-wide DNA methylation alterations.^6^

Despite evidence of differential hepatic tumorigenesis by etiology,^7^^,^^8^ most existing studies on DNA methylation profiles for HCC detection have been focused on all-cause HCC.^9^, ^10^, ^11^, ^12^, ^13^ Few studies have assessed HCC detection in patients with viral hepatitis^14^^,^^15^ or all-cause liver cirrhosis.^16^^,^^17^ Genetically engineered mouse models with metabolic dysfunction-associated steatohepatitis (MASH)-associated HCC suggest a distinct DNA methylation profile for the progression of MASH to HCC in murine models.^18^^,^^19^ Existing human studies on MASH-associated HCC have focused on liver tissues, comparing DNA methylation status of tumor samples to paired adjacent noncancer tissues or to noncancer liver tissues from different individuals, but these have rarely been validated in circulating blood for noninvasive testing because of difficulty in obtaining appropriate patient samples.^20^^,^^21^ Identifying promising blood-based DNA methylation markers that could be combined with current clinical markers of HCC (eg, alpha-feto protein [AFP], lectin-reactive AFP [AFP-L3], and des-γ-carboxy prothrombin [DCP]) could enhance clinical surveillance through noninvasive screening for metabolic HCC.

The goal of this study was to perform an epigenome-wide DNA methylation profiling in patients with metabolic HCC and in cancer-free control patients with metabolic liver disease. Our primary aim was to identify differentially methylated 5′-C-phosphate-G-3' (CpG) positions across the genome that discriminate metabolic HCC cases from metabolic controls using blood leukocyte DNA samples. We also sought to develop and validate a multifactorial model combining CpGs with selected clinical and demographic variables. In secondary analysis, we assessed whether presence of the genetic risk variant *PNPLA3* (I148M) rs738409 could further improve metabolic HCC prediction by combining this variant with differentially methylated CpGs and clinical and demographic variables.

## Materials and Methods

### Study Population and Data Collection

Details of the design and methods used for participant recruitment and data collection have been published.^22^ Briefly, data and biospecimen were obtained from the following six international sites: 1) the Barcelona Clinic Liver Cancer Group (BCLC), Hospital Clinic Barcelona, and IDIBAPs, Barcelona, Spain; 2) Instituto de Investigación Sanitaria Biogipuzkoa (IISB), Donostia University Hospital, San Sebastian, Spain; 3) the Karolinska University Hospital, Sweden; 4) the Virgen del Rocio Hospital Institute of Biomedicine of Sevilla (IBIS), Seville, Spain; 5) the University of Texas Southwestern (UTSW), San Antonio, Texas; and 6) the Mayo Clinic sites in Rochester, Minnesota, and Jacksonville, Florida. All sites provided germline leukocyte DNA and epidemiological data on 673 metabolic HCC cases and 763 cancer-free controls with a history of MASLD (formerly known as nonalcoholic fatty liver disease), metabolic syndrome, or other metabolic conditions (eg, diabetes and obesity). The participating sites were asked to exclude individuals with competing liver diseases before submitting their data and DNA samples to the Mayo Clinic. Potential participants excluded from the study included those with at least one of the following liver diseases: viral hepatitis (HBV, HCV), alcoholic liver disease, autoimmune hepatitis, alpha-1-antitrypsin deficiency, hemochromatosis, Wilson’s disease, biliary cirrhosis, primary sclerosing cholangitis, Budd-Chiari syndrome, and those who consumed ≥20 grams of alcohol per day. After these exclusions, metabolic HCC was defined as an imaging or pathologically confirmed diagnosis of steatosis-related HCC, metabolic syndrome-related HCC, or cryptogenic HCC—most of which are associated with MASLD.^23^ Controls were cancer-free individuals with imaging or pathological confirmation of hepatic steatosis. Data received from each site included information on case-control status, age at HCC diagnosis or recruitment for controls, sex, ethnicity, body mass index (kg/m^2^), smoking history, and type II diabetes mellitus. For the present study, we frequency matched 320 metabolic HCC cases with 320 metabolic controls based on age (±5 years), sex, and study site. All participating sites obtained approval from their local institutional review boards (IRBs), and an additional IRB approval was obtained from the Mayo Clinic for the present study (IRB#: 23-000005).

### DNA Methylation Assay and Quality Control Checks

Peripheral blood leukocyte DNA samples obtained from the participants were assayed on the Illumina Infinium Methylation EPIC BeadChip microarray (EPIC array; Illumina Inc, San Diego, CA), which covers over 850,000 CpG sites across the genome.^24^ The assay was performed at the Mayo Clinic Genome Analysis Core laboratory. In brief, DNA quantification was performed using the Invitrogen Qubit dsDNA Quantification Assay kit (catalog #Q32853; ThermoFisher Scientific, Inc, Waltham, MA). This was followed by a bisulfite modification process that utilized the column cleanup kit method under the alternative incubation conditions recommended by Illumina for the EPIC array. Measurements were done on a nanodrop instrument following the bisulfite modification. We ran the EPIC array using eight 96-well plates containing DNA from the 640 cases and controls. We included 16 laboratory control DNA samples (human methylated and unmethylated control DNA sets; catalog #D5011 for methylated and #D5014 for unmethylated control DNA, Zymo Research Inc, Irvine, CA). A pair of these methylated and unmethylated laboratory controls were included on each of the eight plates to determine if any of the probes should be excluded due to poor performance. Further, we included 64 participant duplicate samples that were distributed evenly across the plates. Determination of the methylation status of the target CpG sites involved comparing the ratio of a fluorescent signal from the methylated allele to the sum of the fluorescent signals from both methylated and unmethylated alleles (ie, the β value). The β value per CpG ranges from 0 (unmethylated) to 1 (fully methylated). Both the laboratory controls and participant duplicates indicated excellent assay performance. The unmethylated laboratory controls showed an intraclass correlation of 0.95, while the methylated controls had a correlation of 0.83. For duplicates, we achieved correlations ≥0.98, and we retained the duplicated sample with the highest call rate in the final analysis. For further quality control (QC), CpGs were excluded if they were located at a single nucleotide polymorphism location, failed in more than 10% of samples, were located on the X and Y chromosomes, were determined to be cross-reactive, or overlapped with genetic variants.^24^ This resulted in 691,187 CpGs passing QC. Data were normalized with *dasen* (*dasen* command in *watermelon* R package) that utilizes quantile normalization to normalize methylated and unmethylated intensities separately and address type I and type II probes separately.^25^ A small fraction of missing β values (<0.01%) were imputed using *champ.impute* function with k-nearest neighbor and *k* parameter as five in the *ChAMP* R package. We used principal component analysis to assess batch effect across the eight experimental plates. The principal component analysis was performed on the top 2000 most variable autosome CpG probes, considering all samples (CpG probes with the largest standard deviations in M-values). We then used the Kruskal-Wallis rank-sum test to investigate the association between the top two principal components and the experimental plates, which did not show any association, ruling out batch effect as a concern. To account for differences in leukocyte cell types, we estimated cell type proportions for CD4 T cells (CD4T), CD8 T cells (CD8T), natural killer cells (NK-cells), B lymphocytes (B-cells), monocytes, and neutrophils using a customized set of probes obtained from identifying optimal libraries (IDOL) optimization for blood as implemented in the *FlowSorted.Blood.EPIC* Bioconductor package.^26^ For participant samples QC, we excluded samples with (1) poor assay performance based on the methylated and unmethylated intensity plot, (2) samples that failed biological sex check using the methods implemented in the *minfi* and *watermelon* R packages, and (3) samples determined to be outliers based on the *watermelon* method.^25^ The underlying methylation data, along with key biological variables (age and sex), have been made publicly available in the NCBI GEO database (GSE281691).^27^

### Statistical Analysis

Differences in participant characteristics were compared using means and standard deviations (SDs) for continuous variables and frequencies and percentages for categorical variables. The study sample was divided randomly into training (80%) and validation (20%) sets through a stratified approach based on frequencies that ensured approximately equal distributions by case-control status, age (5-year groups), sex, and study site in both the training and validation data. We assessed differences in the distribution of all study variables between the cases and controls in the training and validation data separately, but conclusions were based on results of the training data. The variables examined are age (continuous), sex, race (White, other), body mass index (continuous), smoking history (never, former, current), diabetes mellitus (yes, no), study site (Mayo Clinic and UTSW combined, Karolinska hospital, BCLC-Barcelona and IISB-San Sebastian combined, and IBIS-Seville), and leukocyte cell type (CD4T, CD8T, NK-cells, B-cells, monocytes, and neutrophils). These comparisons were done using a Kruskal-Wallis rank-sum test for continuous variables and χ^2^ test for categorical variables. We combined data from UTSW with Mayo Clinic data because the UTSW data comprised only case subjects, and the IISB-San Sebastian data were combined with the BCLC-Barcelona data because the IISB data also comprised case subjects only. Variables found to be significantly different between cases and controls in the training data are race, diabetes mellitus, CD4T, monocytes, and neutrophils, and these were considered for further evaluation as covariates for (1) CpG selection (significant cell types) or (2) multifactorial prediction modeling (race and diabetes).

Candidate CpG selection and initial predictive modeling were done in the training data. Of the 691,187 CpGs that passed the QC checks, we used false-discovery rate-corrected *P*-value (*q*-value), adjusting for the three significant cell types (CD4T, monocytes, and neutrophils), to identify 164 differentially methylated CpGs that met the significance threshold (*q* < 0.05) (Table A1). These CpGs were identified by comparing the metabolic HCC cases with the metabolic controls in the training data and using the moderated paired *t*-test from the R Bioconductor package, linear models for microarray data (*limma*).^28^ To address high dimensionality and multicollinearity among the selected CpGs, least absolute shrinkage and selection operator (LASSO) regression with 10-fold cross-validation was employed using a generalized linear model via penalized maximum likelihood (*glmnet*). The grid search in *glmnet* involved keeping the alpha value fixed at one and varying lambda (regularization parameter) values. The LASSO regression process generated shrunken estimates for each CpG, and we retained only 55 CpGs with nonzero coefficients for prediction modeling, as these are the most informative markers. We used a Manhattan plot to visualize the CpGs across chromosomes and a volcano plot to visualize the hypomethylated and hypermethylated CpGs. Methylation values of the CpGs were also visualized using heatmaps. These data visualizations were done using the R packages *ggplot2* and *ComplexHeatmap*.

In our primary analysis, we first constructed a predictive model that included key biological variables (age and sex) and the significant demographic and clinical variables described above (race and diabetes) using area under the receiver operating characteristic curve (AUC-ROC) analysis with the R package *pROC.* This initial model was constructed to provide a baseline context for evaluating the predictive value of the identified CpGs. We followed this with a predictive model that included only the parsimonious list of 55 differentially methylated CpGs using AUC-ROC analysis. We then constructed a multifactorial model that combined the key biological-demographic and clinical variables (age, sex, race, and diabetes) with the 55 differentially methylated CpGs in the same model to evaluate the performance of an elaborate model and compare it with the performance of the CpGs-only model. We performed two secondary analyses. In the first secondary analysis, we evaluated the additional predictive impact of *PNPLA3*-rs738409 in a subgroup of participants with genetic data available from our previous study.^22^ Here too, we constructed a base model that included only the demographic and clinical variables (age, sex, race, and diabetes mellitus) and rs738409 using AUC-ROC analysis. This was also followed by a separate model for only the 55 CpGs in this subgroup of participants. We then constructed an elaborate model that included age, sex, race, diabetes mellitus, rs738409, and the 55 CpGs. All training data predictive models underwent validation in an independent 20% of the sample, evaluating AUC, sensitivity, and specificity. In the second secondary analysis, we built similar predictive models using only the hypermethylated CpGs.

## Results

Of the 640 participant samples included in the study, one sample showed poor assay performance, 46 samples had discordance between self-reported sex and biological sex inferred from the X:Y chromosome, and five samples were identified as outliers. After excluding these samples, 588 samples remained for analyses (272 metabolic HCC cases and 316 metabolic controls) (Table 1). Briefly, in the overall sample, there was a greater representation of men (65%), non-Hispanic Whites (87%), and individuals with type II diabetes mellitus (65%). Data on the *PNPLA3*-rs738409 genetic risk variant were available on 75% (n = 439) of participants. We split the overall sample in an 80:20 ratio into training (n = 469) and validation (n = 119) sets, respectively. The cases and controls did not differ significantly by age, sex, or study site in either the training or validation sample (Table 1). In the training data, cases had greater proportions of non-Whites and individuals with a history of type II diabetes mellitus than controls. The case participants also had higher leukocyte proportions of CD4T, monocytes, and neutrophils than did the controls. There were no other significant differences observed in the training data. In the validation data, only non-Whites, individuals with diabetes mellitus, and those with a higher monocyte cell type proportions were higher in cases than controls.

**Table 1:** Descriptive Statistics of the Training and Validation Samples

Characteristics	Overall sample *N* = 588 (100%)	Training sample *N* = 469 (80%)			Validation sample *N* = 119 (20%)		
		Cases *N* = 219	Controls *N* = 250	*P*-value	Cases *N* = 53	Controls *N* = 66	*P-*value
Age in y, mean (SD)^a^	64.8 (10.8)	64.8 (12.1)	64.7 (10.6)	.91	65.3 (8.1)	65.1 (8.7)	.94
BMI in kg/m^2^, mean (SD)	31.8 (6.8)	31.2 (5.8)	32.4 (7.3)	.07	30.7 (4.3)	32.2 (8.9)	.27
	*N* (%)	*N* (%)	*N* (%)		*N* (%)	*N* (%)	
Sex				.50			.85
Male	385 (65.5)	145 (66.2)	158 (63.2)		37 (69.8)	45 (68.2)	
Female	203 (34.5)	74 (33.8)	92 (36.8)		16 (30.2)	21 (31.8)	
Race/Ethnicity				<.01			.01
Non-hispanic white	512 (87.1)	174 (79.5)	232 (92.8)		43 (81.1)	63 (95.5)	
Other	76 (12.9)	45 (20.5)	18 (7.2)		10 (18.9)	3 (4.5)	
Smoking history				.65			.06
Never	287 (48.8)	102 (46.6)	123 (49.2)		24 (45.3)	38 (57.6)	
Former smoker	258 (43.9)	104 (47.5)	105 (42.0)		22 (41.5)	27 (40.9)	
Current smoker	34 (5.8)	13 (5.9)	14 (5.6)		6 (11.3)	1 (1.5)	
Unknown	9 (1.5)	0 (0)	8 (3.2)		1 (1.9)	0 (0)	
Type II diabetes mellitus				<.001			.01
Yes	381 (64.8)	171 (78.1)	130 (52.0)		42 (79.2)	38 (57.6)	
No	207 (35.2)	48 (21.90)	120 (48.0)		11 (20.8)	28 (42.4)	
Study site				.85			.40
Mayo Clinic, MN and FL, and UTSW^b^	481 (81.8)	172 (78.5)	199 (79.6)		49 (92.5)	61 (92.4)	
Karolinska university Hospital, Sweden	46 (7.8)	20 (9.1)	22 (8.8)		3 (5.7)	1 (1.5)	
BCLC, Barcelona, and IISB, San Sebastian, Spain	36 (6.1)	17 (7.8)	15 (6.0)		1 (1.9)	3 (4.5)	
IBIS, Seville, Spain	25 (4.3)	10 (4.6)	14 (5.6)		0 (0.0)	1 (1.5)	
White blood cell types							
CD4 T cells, mean (SD)	0.14 (0.07)	0.14 (0.09)	0.16 (0.06)	<.001	0.12 (0.05)	0.14 (0.07)	.12
CD8 T cells, mean (SD)	0.07 (0.05)	0.07 (0.06)	0.07 (0.05)	.19	0.07 (0.05)	0.14 (0.07)	.63
Natural killer cells, mean (SD)	0.05 (0.03)	0.05 (0.03)	0.05 (0.03)	.06	0.05 (0.03)	0.05 (0.02)	.08
B lymphocytes, mean (SD)	0.05 (0.04)	0.05 (0.04)	0.05 (0.03)	.84	0.06 (0.05)	0.04 (0.02)	.09
Monocytes, mean (SD)	0.09 (0.04)	0.10 (0.06)	0.09 (0.03)	.005	0.10 (0.04)	0.08 (0.03)	.01
Neutrophils, mean (SD)	0.59 (0.15)	0.60 (0.19)	0.59 (0.11)	.002	0.61 (0.14)	0.61 (0.12)	.38
*PNPLA3*-rs738409 genotype				0.16			.94
CC	150 (25.5)	54 (24.7)	68 (27.2)		14 (26.4)	14 (21.2)	
CG	173 (29.4)	71 (33.8)	66 (26.4)		17 (32.1)	19 (28.8)	
GG	116 (19.7)	50 (22.8)	37 (14.8)		15 (28.3)	14 (21.2)	
Missing	149 (25.3)	44 (20.1)	79 (31.6)		7 (13.2)	19 (28.8)	

BCLC, Barcelona Clinic Liver Cancer Group, Barcelona, Spain; BMI, body mass index; IBIS, Institute of Biomedicine of Sevilla, Seville, Spain; IISB, Instituto de Investigación Sanitaria Biodonostia Research Institute, Donostia University Hospital, San Sebastian, Spain; UTSW, University of Texas Southwestern.

a Age at hepatocellular carcinoma diagnosis for cases and age at recruitment for controls.

b Data from the UTSW were all cases (N = 43) and therefore were combined with Mayo Clinic samples.

**Figure 1:**
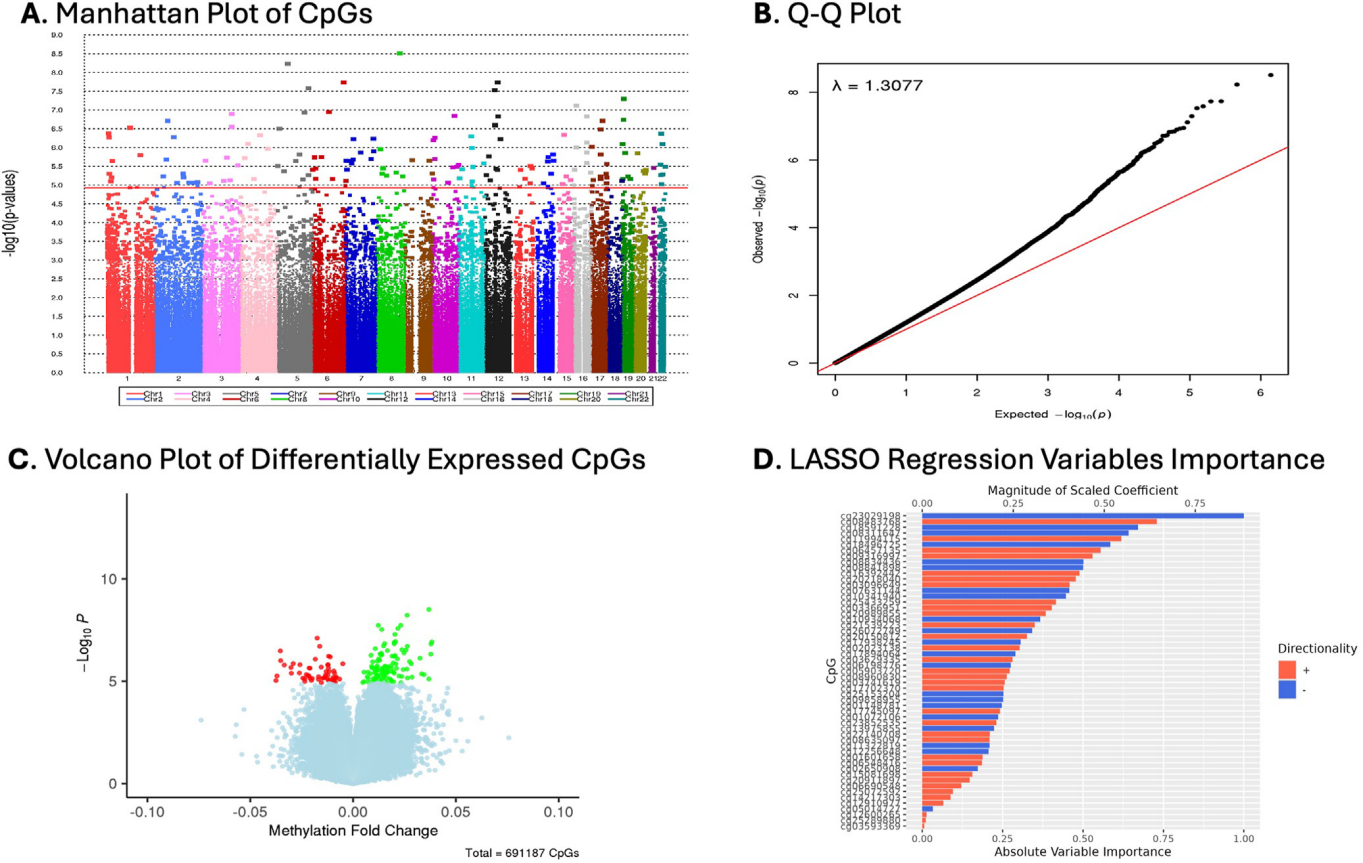
Epigenome-wide analysis for selection of differentially methylated CpGs associated with metabolic HCC. The analysis was performed among 272 metabolic HCC cases and 316 metabolic controls. (A) Manhattan plot with false discovery rate (FDR)-adjusted *P*-value threshold (red horizontal line) for selection of significant CpGs (*q*-value < 0.05; n = 164 CpGs) in the training data for further screening. (B) Q-Q plot of CpGs showing a lambda (λ) value that is closer to 1. (C) Volcano plot of the 164 FDR-significant CpGs, showing hypomethylated CpGs in red color and hypermethylated CpGs in green color among cases vs controls in the training data. (D) Results of a LASSO regression model with 10-fold cross-validation, reducing the 164 FDR-significant CpGs to a parsimonious list of 55 CpGs with nonzero coefficients (33 hypermethylated and 22 hypomethylated) and scaling of absolute importance of each CpG in the presence of the other CpGs. This is the final set of CpGs used for the primary analysis. LASSO, least absolute shrinkage and selection operator.

We performed an epigenome-wide association study (EWAS) in the training data based on 691,187 CpGs that passed QC (Figure 1A-D). The EWAS did not show overfitting as the genomic inflation lambda value is closer to one, which is within the expected range (λ = 1.31, Figure 1B). Of the 691,187 CpG sites, 164 were differentially methylated (110 hypermethylated and 54 hypomethylated) in the metabolic HCC cases compared to the metabolic controls (Figure 1A and C, and Table A1). We used LASSO regression with 10-fold cross-validation to assess multicollinearity and reduced the EWAS significant CpGs to a parsimonious list of 55 informative markers with nonzero coefficients, of which 33 were hypermethylated and 22 were hypomethylated (Figure 1D and Table 2). Interestingly, many of the genes linked to the differentially methylated CpGs have been associated with liver disease progression (eg, *DCP2, TRPV3, ARRB1, KCNIP4, MIR10A*) and cancer formation or progression (eg, *MTHFR, GRIK2, GSN, HOX3, KCNMA1*) (Table 2).

**Table 2:** Differentially Methylated CpGs Used for the Primary Analysis; *N* = 55 CpGs (33 Hypermethylated and 22 Hypomethylated)

CpG probe	Chromosomal position^a^	Genes	Relation to Island	Coefficient	Cases: Mean beta value	Controls: Mean beta value	Log-fold change^b^	Raw *P*-value
cg25433259	Chr6:159271784	*OSTCP1*	OpenSea	5.1082312	0.66541858	0.63835286	0.01226212	1.86E-08
cg16392442	Chr12:49595954	*-*	OpenSea	15.2001542	0.17353326	0.15696093	0.01394544	2.95E-08
cg06457135	chr19:5953406	*RANBP3*	OpenSea	9.3450824	0.40866314	0.38436786	0.02035666	5.09E-08
cg12910977	chr5:138207492	*LRRTM2;CTNNA1*	OpenSea	0.7322945	0.56139306	0.5179095	0.03832187	1.18E-07
cg02023138	chr12:51701979	*BIN2*	OpenSea	11.3546081	0.84181837	0.83196508	0.01081285	2.53E-07
cg07631144	chr17:46657393	*MIR10A*	N_Shore	−4.1056162	0.29359393	0.33761632	−0.0353619	3.28E-07
cg03629335	chr4:100126967	*ADH6*	OpenSea	2.8296323	0.69035821	0.65791421	0.03776824	4.69E-07
cg17745097	chr1:11863365	*MTHFR*	N_Shelf	10.4633105	0.08307216	0.07659769	0.00870919	5.36E-07
cg17938245	chr12:80067352	*PAWR*	OpenSea	−5.5211165	0.68665745	0.693618	−0.0119055	5.92E-07
cg20911897	chr4:37953726	*TBC1D1*	OpenSea	1.9160149	0.46867666	0.45262885	0.02157469	7.96E-07
cg06690548	chr4:139162808	*SLC7A11*	OpenSea	1.8024676	0.78789942	0.76138798	0.0274857	1.07E-06
cg11322819	chr19:13694039	*-*	OpenSea	−14.1189425	0.07830778	0.08513437	−0.0049903	1.38E-06
cg17894064	chr5:112657195	*MCC*	OpenSea	−4.1526787	0.73211569	0.7569463	−0.0258219	1.54E-06
cg22140708	chr6:10081664	*-*	OpenSea	5.1449817	0.78206787	0.77170398	0.0154948	1.83E-06
cg20989855	chr4:20985927	*KCNIP4*	OpenSea	9.0390939	0.22096169	0.20473515	0.0164293	1.90E-06
cg08311647	chr10:11596182	*USP6NL*	OpenSea	−8.2102209	0.32034876	0.33414088	−0.0155017	2.03E-06
cg03096649	chr7:130056977	*CEP41*	OpenSea	13.3323684	0.80181661	0.78461484	0.01153379	2.03E-06
cg09858955	chr2:58135951	*VRK2*	OpenSea	−2.8610325	0.35796606	0.3978885	−0.0302066	2.11E-06
cg05903720	chr14:104663241	*-*	OpenSea	5.7993613	0.67104233	0.65604405	0.01851333	2.23E-06
cg20150812	chr9:124029753	*GSN*	OpenSea	5.1334782	0.63156105	0.6199896	0.01418724	2.25E-06
cg25072592	chr14:75355586	*DLST*	OpenSea	1.5676053	0.53199877	0.51258117	0.0210767	2.34E-06
cg17702370	chr17:79283128	*C17orf55*	N_Shore	8.2558677	0.15774941	0.14859067	0.0119348	2.79E-06
cg20218040	chr6:14369697	*-*	OpenSea	8.2222083	0.78452904	0.76199551	0.01677532	3.60E-06
cg12600265	chr7:5422883	*TNRC18*	OpenSea	0.2650506	0.57937612	0.56715465	0.01639226	3.88E-06
cg10934068	chr6:509762	*EXOC2*	OpenSea	−3.3359868	0.58562148	0.59379713	−0.0261018	3.92E-06
cg01148781	chr8:99963021	*OSR2*	S_Shore	−4.0754728	0.127759	0.14846004	−0.0227403	4.70E-06
cg08483768	chr16:86304619	*-*	OpenSea	8.0038342	0.50945873	0.48237272	0.0344807	4.72E-06
cg01072106	chr9:138952311	*NACC2*	OpenSea	−5.4605017	0.66997388	0.69090851	−0.0133822	4.89E-06
cg09316997	chr14:91874913	*CCDC88C*	OpenSea	6.4844744	0.58855649	0.56312738	0.01150639	5.05E-06
cg02650908	chr17:74889830	*MGAT5B*	OpenSea	−3.0128946	0.7169462	0.73871776	−0.0218802	5.07E-06
cg25289880	chr1:13856326	*-*	OpenSea	0.3631003	0.11750793	0.10623291	0.01184671	5.07E-06
cg08834436	chr22:27831818	*-*	N_Shelf	−5.1226004	0.33084528	0.36666469	−0.0370071	5.43E-06
cg03593369	chr2:43456557	*-*	S_Shore	0.1501873	0.29323094	0.27353325	0.01358761	5.89E-06
cg13975855	chr17:46652550	*HOXB3*	N_Shore	−4.3014449	0.7616477	0.78337037	−0.0167974	5.93E-06
cg06548416	chr1:24438703	*MYOM3*	OpenSea	2.4685847	0.23242444	0.20555792	0.02863055	6.24E-06
cg23029198	chr2:146510791	*-*	OpenSea	−39.0402834	0.79023973	0.79559585	−0.0081623	6.25E-06
cg18591228	chr11:3175552	*OSBPL5*	OpenSea	−8.1292725	0.35349018	0.37448402	−0.0097212	6.57E-06
cg06198776	chr13:73557424	*PIBF1*	OpenSea	−11.514518	0.87081259	0.88156024	−0.008382	6.73E-06
cg26072749	chr17:46657274	*MIR10A*	N_Shore	−6.10247	0.15782119	0.17616999	−0.0143176	6.87E-06
cg11994115	chr19:40360856	*FCGBP*	N_Shore	8.7958448	0.64306667	0.61853271	0.02644958	6.87E-06
cg15081698	chr6:101847050	*GRIK2*	Island	5.0372892	0.12331257	0.11170805	0.01080277	6.97E-06
cg18496725	chr4:68788615	*TMPRSS11A*	OpenSea	−9.0086014	0.75106914	0.77602459	−0.0244003	6.97E-06
cg05014727	chr10:6214016	*PFKFB3*	OpenSea	−0.373298	0.28626944	0.31704808	−0.0198905	7.25E-06
cg23852535	chr17:8857258	*PIK3R5*	OpenSea	10.6771863	0.83787502	0.83078653	0.00737971	7.32E-06
cg14217303	chr15:85177537	*SCAND2P*	S_Shore	3.2816993	0.25645962	0.24704736	0.00983248	7.36E-06
cg12256648	chr3:143752097	*-*	OpenSea	−3.2169829	0.42099641	0.44279351	−0.0234836	7.53E-06
cg08841898	chr12:27717865	*PPFIBP1*	OpenSea	−18.5292437	0.68745191	0.70022304	−0.0092687	7.55E-06
cg10341940	chr18:76822780	*-*	OpenSea	−24.5248737	0.82390723	0.82977233	−0.006565	7.77E-06
cg25153204	chr10:79291246	*KCNMA1*	OpenSea	−7.5939005	0.84999289	0.85835243	−0.0110492	8.58E-06
cg03366951	chr3:39302545	*-*	OpenSea	6.709593	0.16306437	0.15399861	0.01408014	8.95E-06
cg03741619	chr17:3438918	*TRPV3*	Island	16.8968122	0.07389002	0.06818281	0.00544581	1.03E-05
cg01601658	chr6:168785524	*-*	OpenSea	3.3166661	0.30778145	0.288747	0.01968328	1.04E-05
cg08635097	chr13:44833857	*-*	OpenSea	9.6920045	0.12942612	0.12314091	0.00755129	1.11E-05
cg21539223	chr5:112312093	*DCP2*	N_Shore	27.1214227	0.04927896	0.04464676	0.00476079	1.13E-05
cg08960830	chr11:75047180	*ARRB1*	OpenSea	8.8896234	0.69990481	0.69248749	0.00905003	1.16E-05

a Positions are based on the human reference genome assembly GRCh38.

b Fold change comparing beta estimates between cases and controls.

**Figure 2:**
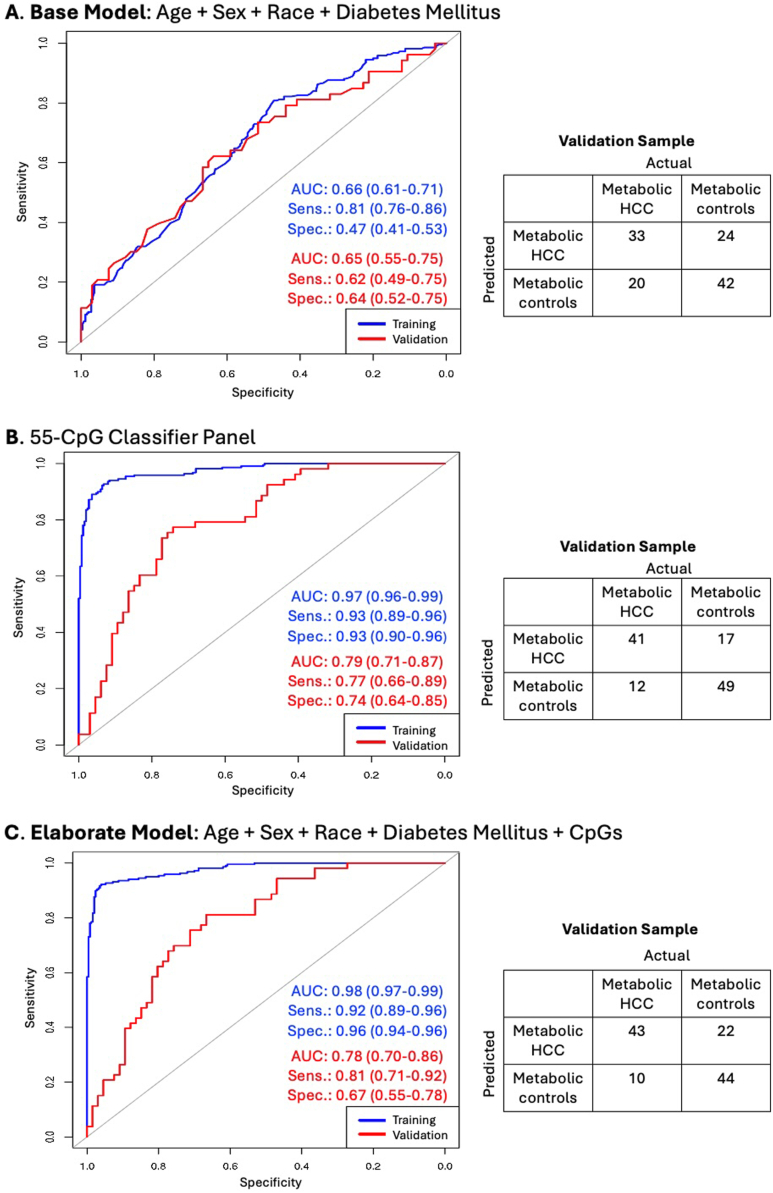
Distinguishing metabolic HCC from benign metabolic liver disease using demographic and clinical variables and differentially methylated CpGs. The study sample comprised 272 metabolic HCC cases and 316 metabolic controls. (A) Training and validation results from area under the receiver operating characteristic curve (AUC-ROC) analysis for a model that included age (continuous), sex, race (White, other), and type II diabetes mellitus (yes, no). (B) AUC-ROC analysis for a model that included only the 55 differentially methylated CpGs as shown in Table 2. (C) An elaborate multifactorial AUC-ROC analysis for a model that included age, sex, race, diabetes mellitus, and the 55 CpGs. AUC, area under the receiver operating curve; HCC, hepatocellular carcinoma; sens., sensitivity; spec., specificity.

To provide a context for assessing the discriminatory accuracy of the 55 informative CpGs, we first created a base model comprising demographic and clinical variables only: age, sex, race, and diabetes mellitus. This base model yielded a training sample AUC = 0.66 (95% confidence interval [CI]: 0.61–0.71), sensitivity = 0.81 (95% CI: 0.76–0.86), and specificity = 0.47 (95% CI: 0.41–0.53) and a validation sample AUC = 0.65 (95% CI: 0.55–0.75), sensitivity = 0.62 (95% CI: 0.49–0.75), and specificity = 0.64 (95% CI: 0.52–0.75) (Figure 2A). Next, we assessed the predictive accuracy of only the parsimonious panel of 55 informative CpGs in the training data, yielding AUC = 0.97 (95% CI: 0.96–0.99), sensitivity = 0.93 (95% CI: 0.89–0.96), and specificity = 0.93 (95% CI: 0.90–0.96) (Figure 2B). The validation results for the CpGs-only model were AUC = 0.79 (95% CI: 0.71–0.87), sensitivity = 0.77 (95% CI: 0.66–0.89), and specificity = 0.74 (95% CI: 0.64–0.85) (Figure 2B). We then assessed the combined predictive ability of an elaborate model that included age, sex, race, diabetes mellitus, and the 55 CpGs, yielding training sample AUC = 0.98 (95% CI: 0.97–0.99), sensitivity = 0.92 (95% CI: 0.89–0.96), and specificity = 0.96 (95% CI: 0.94–0.96). Results from the validation sample for the joint elaborate model were AUC = 0.78 (95% CI: 0.70–0.86), sensitivity = 0.81 (95% CI: 0.71–0.92), and specificity = 0.67 (95% CI: 0.55–0.78) (Figure 2C). These results constitute our primary findings.

**Figure 3:**
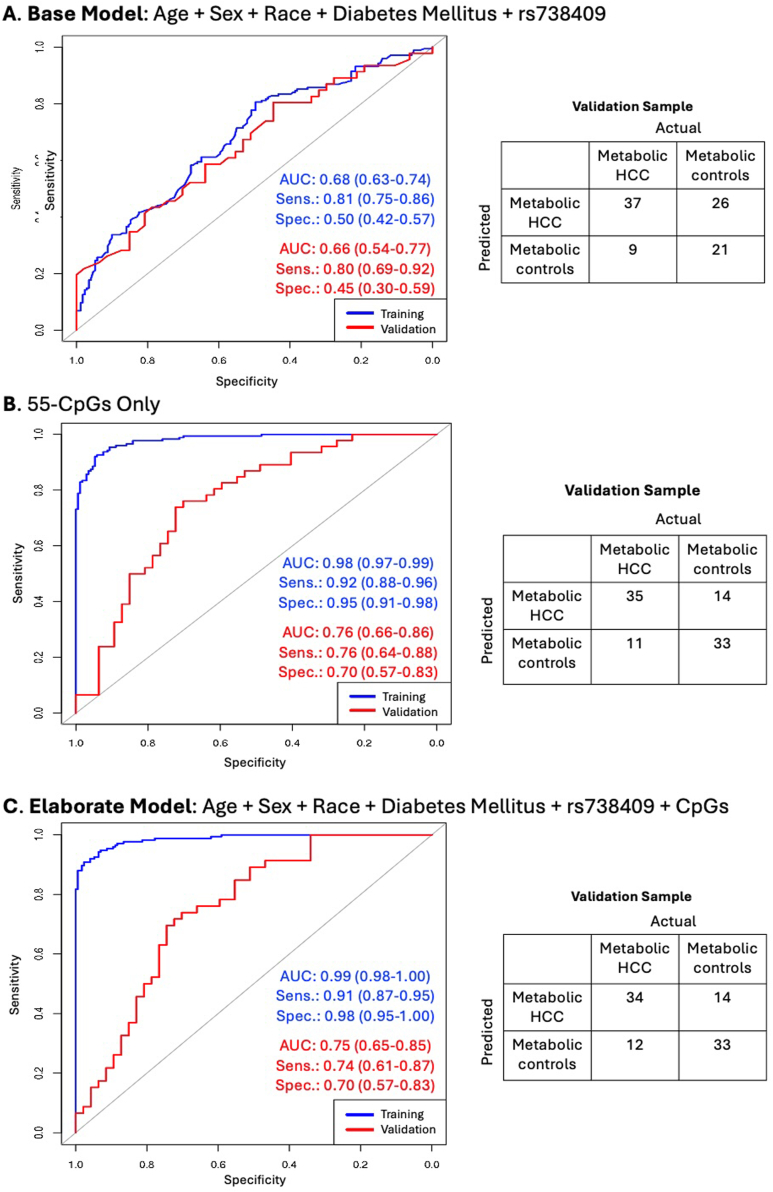
Discriminating between metabolic HCC and metabolic liver disease in a subgroup of participants with genetic data. These analyses were performed among 75% of the study sample (n = 439). (A) Training and validation results from area under the receiver operating characteristic curve (AUC-ROC) analysis for a model that included age (continuous), sex, race (White, other), diabetes mellitus (yes, no), and *PNPLA3*-rs738409 genotype. (B) Training and validation results for a model that included only the 55 differentially methylated CpGs as shown in Table 2. (C) Multifactorial AUC-ROC analysis for metabolic HCC combining the clinical and demographic variables with CpGs. This multifactorial model was built using LASSO regression with 10-fold cross-validation and examining the clinical and demographic variables and the 55 CpGs. However, only 44 CpGs with nonzero coefficients were retained in addition to age, sex, race, diabetes mellitus, and rs738409 for prediction modeling. AUC, area under the receiver operating curve; HCC, hepatocellular carcinoma; sens, sensitivity; spec, specificity.

In secondary analysis among a subgroup of participants with genetic data, we assessed the additional predictive impact of the HCC susceptibility variant, *PNPLA3*-rs738409 (Figure 3). Here too, we created a base model that comprised only age, sex, race, diabetes mellitus, and rs738409, yielding validation sample AUC = 0.66 (95% CI: 0.54–0.77), sensitivity = 0.80 (95% CI: 0.69–0.92), and specificity = 0.45 (95% CI: 0.30–0.59) (Figure 3A). Validation results for a model with only the 55 CpGs in this subgroup were AUC = 0.76 (95% CI: 0.66–0.86), sensitivity = 0.76 (95% CI: 0.64–0.88), and specificity = 0.70 (95% CI: 0.57–0.83) (Figure 3B). Further, we built an elaborate model that assessed the combined predictive ability of the clinical, demographic, and genetic data together with the 55 CpGs in the subgroup of participants with available genetic data. After running a penalized LASSO regression for the elaborate model in this subgroup analysis, only 44 of the CpGs had nonzero coefficients (Table A2), and together with age, sex, race, diabetes mellitus, and rs738409, they were used for prediction modeling. This elaborate model yielded a validation AUC = 0.75 (95% CI: 0.65–0.85), sensitivity = 0.74 (95% CI: 0.61–0.87), and specificity = 0.70 (95% CI: 0.57–0.83) Figure 3C).

**Figure 4:**
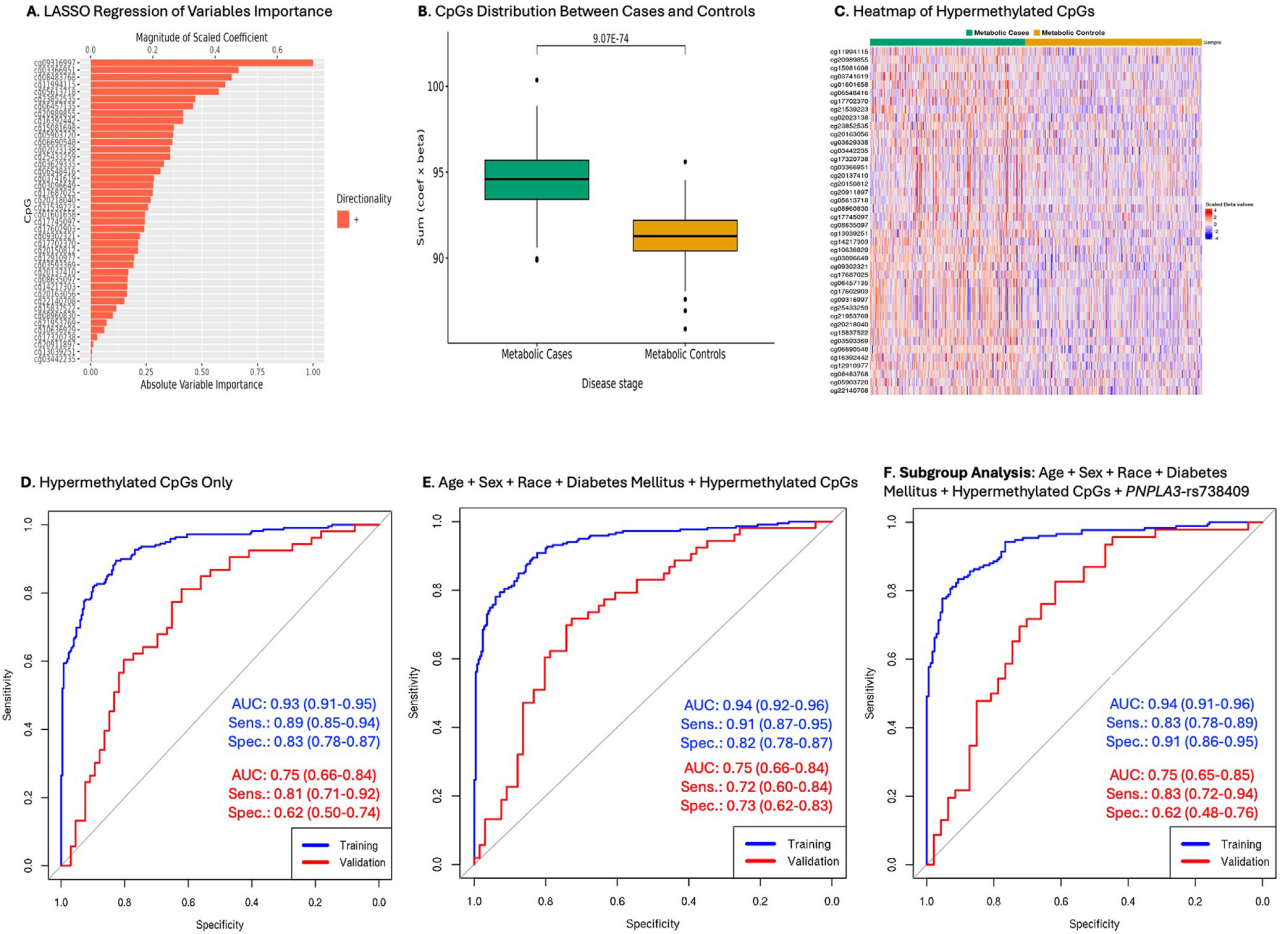
Characterizing metabolic HCC using hypermethylated CpGs only and in combination with clinical, demographic, and *PNPLA3*-rs738409. The analysis was performed among 272 metabolic HCC cases and 316 metabolic controls. (A) LASSO regression with scaled absolute importance of 42 hypermethylated CpGs used for the CpGs-only model. (B) Differential distribution of the combined product of the 42 hypermethylated CpGs (estimated coefficients x beta values) between cases and controls. (C) Heatmap of 42 selected CpGs in the training data. (D) Modeling of area under the receiver operating characteristic curves (AUC-ROCs) for the hypermethylated CpGs only (n = 42) in the training and validation samples. (E) A separate model that evaluated the combination of age (continuous), sex, race (White, other), type II diabetes mellitus (yes, no), and the hypermethylated CpGs in a distinct LASSO regression model with 10-fold cross-validation, retaining 40 hypermethylated CpGs plus age, sex, race, and diabetes for prediction modeling. (F) A subgroup analysis modeling AUCs for the hypermethylated CpGs plus age, sex, race, diabetes, and *PNPLA3*-rs738409 among participants with genetic data (n = 439) using a separate LASSO regression with 10-fold cross-validation. This analysis retained 38 CpGs, age, sex, race, diabetes, and rs738409 for prediction modeling in the training (n = 346) and validation (n = 93) samples. AUC, area under the receiver operating curve; HCC, hepatocellular carcinoma; sens., sensitivity; spec., specificity.

We repeated all analyses using only the hypermethylated CpGs from the EWAS significant CpGs (n = 110, *q* < 0.05). Based on a penalized LASSO regression analysis with 10-fold cross-validations, we identified a 42-CpG classifier panel with nonzero coefficients that showed differential methylation values between cases and controls (Figure 4A–C and Table A3). Upon fitting the 42 hypermethylated CpGs, we observed validation AUC = 0.75 (95% CI: 0.66–0.84), sensitivity = 0.81 (95% CI: 0.71–0.92), and specificity = 0.62 (95% CI: 0.50–0.74) (Figure 4D). We performed a separate multifactorial penalized LASSO regression analysis that included age, sex, race, diabetes mellitus, and the 42 CpGs, retaining 40 CpGs with nonzero coefficients (Table A4) together with age, sex, race, and diabetes mellitus. This yielded a validation AUC = 0.75 (95% CI: 0.66–0.84), sensitivity = 0.72 (95% CI: 0.60–0.84), and specificity = 0.73 (95% CI: 0.62–0.83) (Figure 4E). We further constructed an independent model in the subgroup of participants with genetic data, fitting a penalized LASSO regression analysis with the 42 hypermethylated CpGs, retaining 38 CpGs (Table A5) together with age, sex, race, diabetes mellitus, and rs738409 for prediction modeling. This resulted in a validation AUC = 0.75 (95% CI: 0.65–0.85), sensitivity = 0.83 (95% CI: 0.72–0.94), and specificity = 0.62 (95% CI: 0.48–0.76) (Figure 4F).

## Discussion

In this large multicenter international study, we performed the EWAS in patients with metabolic liver disease from which 55 differentially methylated CpGs were identified and independently validated for association with metabolic HCC. To provide a context for evaluating the predictive accuracy of the identified CpGs, we first constructed a base model that comprised age, sex, race, and diabetes mellitus, yielding a validation AUC of 0.65, sensitivity of 0.62, and specificity of 0.64. This base model did not perform as well as our 55-CpG classifier model with a validation AUC of 0.79, sensitivity of 0.77, and specificity of 0.74. We also developed a multifactorial model that combined age, sex, race, and diabetes mellitus with the 55-CpG panel, and this elaborate model had slightly higher sensitivity but lower specificity in the validation sample (AUC = 0.78, sensitivity = 0.81, specificity = 0.67) compared to the 55-CpGs-only model. Further, we explored a multifactorial model in a subgroup of participants with genetic data, jointly assessing the predictive accuracy of age, sex, race, diabetes mellitus, *PNPLA3*-rs738409, and the 55 CpGs. Validation results of this model (AUC = 0.75, sensitivity = 0.74, specificity = 0.70) did not differ substantially from a model built with only the 55 CpGs in the same subgroup of participants (AUC = 0.76, sensitivity = 0.76, specificity = 0.70). Together, the sensitivity values of these models are higher or nearly at par with reported sensitivity of AFP, the most widely used HCC diagnostic marker, with published sensitivity values of AFP ranging between 0.48 and 0.84 for the detection of all-cause HCC.^14^^,^^15^ However, because 20%–30% of HCC tumors do not secrete AFP,^29^ future studies that combine relevant CpGs with AFP or other clinical diagnostic markers (eg, DCP) and genetic risk variants for multifactorial modeling could enhance prediction of HCC in patients with metabolic liver disease.

DNA methylation plays an important role in transcriptome regulation and gene expression.^30^ Aberrant DNA methylation has been found to be stably maintained by the DNA methyltransferase genes, *DNMT1, DNMT3A,* and *DNMT3B,* during multistage tumorigenesis of various malignancies.^21^^,^^30^ Tumor suppressor gene silencing through DNA hypermethylation and oncogene activation through DNA hypomethylation can both contribute to cancer development, and these methylation markers could become potential targets of therapy.^31^^,^^32^ In hepatic tumorigenesis, aberrant DNA methylation has been observed in the development of HCC, but most methylation studies have focused on a single gene locus,^33^ a target candidate gene panel,^9^^,^^34^^,^^35^ or even all-cause HCC,^9^, ^10^, ^11^, ^12^, ^13^ but these have not proven to be sufficiently robust when compared to AFP or other clinical diagnostic biomarkers. A meta-analysis of 20 studies on all-cause HCC found that target candidate gene-based CpG panels do not perform adequately well to inform clinical test development.^36^ Our use of an unbiased EWAS approach for screening of informative markers has the advantage of identifying potentially novel methylation markers for etiology-specific HCC detection, which is important for metabolic HCC given evidence of its distinct molecular signatures,^6^ and its fast-rising incidence worldwide.^1^^,^^2^

Because aberrant methylation can repress tumor suppressor genes or enhance oncogene activity,^31^^,^^32^ it is important to assess the effects of both hypermethylated and hypomethylated CpGs jointly regarding tumor development. In our primary analysis, we identified 33 hypermethylated and 22 hypomethylated CpGs that play potential roles in metabolic HCC development (Table 2). Among the genes linked to hypermethylation in the cases, *TRPV3*,^37^ *DCP2*,^38^ *KCNIP4*,^39^ and *ARRB1*^40^ have been associated with progression of liver disease to fibrosis and cirrhosis. Upregulation of *ARRB1* has been further found to induce inflammation-associated HCC development, while inhibition of this gene reduces hepatic inflammation and tumorigenesis.^40^ Other studies have found higher expression of *ARRB1* during HCC metastasis,^41^ and its upregulation correlates with tumor progression.^42^ *MTHFR* is another hypermethylated CpG-linked gene found in this study, and polymorphisms in this gene, which is involved in one-carbon metabolism of folate, have been associated with higher HCC risk and poor prognosis of HCC patients.^43^^,^^44^ *GRIK2* has been associated with liver cancer development and metastasis.^45^ *In vivo* experiments have shown that overexpression of *GSN,* another hypermethylated CpG-linked gene, promotes HCC development through inhibition of the *TP53* tumor suppressor gene.^46^ *GSN* has also been found to promote HCC invasion and metastasis through its regulation of epithelial-mesenchymal transition.^47^^,^^48^

Among the hypomethylated genes, a study by Wang *et al* suggests that *HOXB3,* which is involved in cell growth and differentiation, is downregulated in cryptogenic HCC development^49^ and also downregulated in breast and pancreatic cancers.^50^ In another study, *HOXB3* was found to interact with *DNMT3B* to promote leukemia development.^51^ *MIR10A* has been proposed as a marker for liver fibrosis development in chronic liver disease^52^ and found to promote HCC cell proliferation, migration, and metastasis.^53^ Two other hypomethylated CpG-linked genes, *VRK2* and *MGAT5B*, have been associated with HCC metastasis.^54^^,^^55^ *KCNMA1* has been found to be downregulated in HCC, and its upregulation enhances HCC cell lines’ responsiveness to treatment with sorafenib.^56^ Further, *OSBPL5* is reported to be downregulated in HCC.^57^ Although downregulation of *PAWR* has been found to induce bladder cancer, its upregulation with self-amplifying RNA (saRNA) inhibits cancer cell proliferation by inducing apoptosis.^58^ The potential impact of the other genes listed in Table 2 has not been studied extensively and therefore requires further investigation.

Although HCC is typically diagnosed based on clinical, imaging, and/or pathological features, in the present study, we did not aim to establish a diagnostic criterion for metabolic HCC but rather identify DNA methylation markers that can robustly discriminate metabolic HCC from benign metabolic liver disease. Our aim is that these markers could be combined with clinical biomarkers in future studies to improve diagnosis of HCC in patients with chronic metabolic perturbations, including improving diagnosis in patients with asymptomatic disease. Identifying DNA methylation markers that can discriminate between cancer and noncancer samples is an important first step in cancer detection in high-risk patients.^20^ However, whether the markers identified here are aberrantly methylated in the precancer stage or early cancer development stage of the multistage hepatic tumorigenesis would need to be investigated further before the establishment of a specific criterion for metabolic HCC detection. The identified markers have prospects for clinical translation if confirmed in prospective studies with long-term follow-up and with evaluation of early-stage HCC in the background of metabolic liver disease. Since the methylation markers could be targeted with pyrosequencing or high-performance liquid chromatography, both of which can be done in a clinical laboratory, we expect that their clinical application would be feasible.

Our study has several strengths and limitations. Strengths include the focus on patients with metabolic liver disease with well-characterized samples sourced through our multicenter international collaboration. We used the 850k EPIC array for screening of differentially methylated CpGs across the genome, as opposed to the smaller 450k array with limited CpG coverage or targeted assay panels that have been used in prior studies.^16^^,^^17^^,^^20^^,^^21^^,^^32^^,^^59^ Our sample size was sufficiently large and enabled separate training and independent validation analyses. To ensure rigor and reduce redundancy in CpG selection, we employed LASSO regression with 10-fold cross-validations in the training models, which adds to the study’s strengths. Our validation analysis shows the robustness of the models and supports a role of the identified markers in metabolic HCC development. Additionally, we built a separate model focused on only hypermethylated CpGs, which has been done in some studies, but our primary focus was on the combined effect of both the hypermethylated and hypomethylated CpGs.

Limitations of the study include our use of leukocyte DNA samples instead of plasma-derived cell-free DNA (cfDNA) for the methylation assay. While we did not have sufficient plasma volume on our patients for the cfDNA assay, we ameliorated this challenge by estimating leukocyte cell type proportions in each participant sample and adjusted for significant cell types in the model used for selecting differentially methylated CpGs. Importantly, the main differences between cfDNA and leukocyte DNA are that cfDNA captures extracellular fragmented DNA circulating freely in the bloodstream, whereas leukocyte DNA is extracted directly from white blood cells and is intact and intracellular. These approaches can be complementary for DNA methylation-based biomarker discovery, and their differences and complementary application are covered more thoroughly elsewhere.^60^, ^61^, ^62^ We also did not have data on cirrhosis status or tumor stage, and we could not assess these in the study. The cross-sectional nature of our data cannot preclude reverse causality of the association where the presence of a tumor could alter methylation status. However, such alterations could be useful for early HCC detection if confirmed in longitudinal studies. Further, our study sample is predominantly non-Hispanic White. Follow-up studies in a more diverse patient population, preferably using cfDNA and including data on cirrhosis and tumor stage and with larger patient samples, would be an improvement.

In summary, we performed an unbiased epigenome-wide screening of differentially methylated markers using germline leukocyte DNA and identified a promising set of CpGs that can discriminate patients with metabolic HCC from cancer-free patients with metabolic liver disease. These markers could aid in HCC surveillance in patients with metabolic perturbations. Although further work is needed to confirm the markers identified here, they could serve as components of an integrative panel that could ultimately improve outcomes for patients with this frequent deadly cancer.
